# Experience of Pregnancy Loss Among Maternal Nurses and Midwives: A Phenomenological Study

**DOI:** 10.1002/nop2.70892

**Published:** 2026-10-12

**Authors:** Sawsan Abuhammad, Shaher Hamaideh, Hossam Alhawatmeh, Rowaida AlMaaitah, Heba H. Hijazi, Maryam K. El‐zubi, Amat Al‐Khaleq Mehrass, Ahlam Hamaidi, Vidya Seshan, Nabeel Al Yateem

**Affiliations:** ^1^ Department of Nursing College of Health Sciences, University of Sharjah Sharjah UAE; ^2^ Department of Maternal Health Faculty of Nursing, Jordan University of Science and Technology Irbid Jordan; ^3^ Department of Community and Mental Health Nursing Faculty of Nursing, the Hashemite University Zarqa Jordan; ^4^ Department of Adult Health Faculty of Nursing, Jordan University of Science and Technology Irbid Jordan; ^5^ Department of Community and Mental Health Nursing Jordan University of Science and Technology Irbid Jordan; ^6^ Department of Health Services College of Health Sciences, University of Sharjah Sharjah UAE; ^7^ Department of Health Management and Policy Faculty of Medicine, Jordan University of Science and Technology Irbid Jordan; ^8^ Department of Talent Development The International Medical Company Doha Qatar; ^9^ Faculty of Medicine, Dhamar University Dhamar Yemen; ^10^ Department of Community Medicine Faculty of Medicine, Jordan University of Science and Technology Irbid Jordan

**Keywords:** descriptive phenomenology, Jordan, midwives, nurses, pregnancy loss, workplace support

## Abstract

**Aim:**

This study explored the lived experience of pregnancy loss among maternal nurses and midwives in Jordan, with attention to how loss shaped work, grief, and decisions about future pregnancy within the Jordanian cultural and religious context.

**Design:**

Descriptive phenomenological qualitative study.

**Methods:**

Twenty maternal nurses and midwives who had experienced pregnancy loss participated in individual semi‐structured interviews conducted in private locations selected by participants. Snowball sampling was used. Interviews lasted 45–60 min, were audio‐recorded with participants' consent, transcribed, and coded manually by the research team using Colaizzi's seven‐step descriptive phenomenological method. Two researchers coded the data independently before discussing and agreeing on themes.

**Results:**

Participants were 26–55 years old, 17 of 20 had more than 10 years of nursing experience, 17 of 20 worked in health centers, and 18 of 20 had children. Four interpretive themes were developed: loss without warning; embodied loss: inseparable physical and psychological suffering; grieving while caregiving: negotiating work, disclosure, and peer support; and trying again with fear.

**Conclusion:**

Pregnancy loss among maternal nurses and midwives in Jordan produced physical, emotional, occupational, and culturally situated consequences. The study contributes evidence that clinicians who provide maternity care may return to the same clinical spaces that mirror their loss, making workplace flexibility, trusted peer support, and culturally sensitive acknowledgment of grief especially important.

**Public Contribution:**

No patient or public contribution.

## 1 Introduction

Maternal nurses and midwives may experience pregnancy loss not only as women and mothers, but also as clinicians whose work places them in daily contact with pregnancy, birth, and perinatal complications. Existing studies describe pregnancy after perinatal loss, women's experiences of early pregnancy loss, and nurses' encounters with fetal loss in clinical practice (DeBackere et al. 2008; Hutti et al. 2016; Jonas‐Simpson et al. 2013; Nash et al. 2018; Radford and Hughes 2015). These studies show that pregnancy loss can alter expectations for future pregnancy, intensify grief, and shape caregiving encounters; however, they provide limited evidence about clinicians who themselves experience pregnancy loss while continuing to work in maternal settings.

Prior research has shown that perinatal loss can be psychologically distressing for women and families (Bansen and Stevens 1992; Côté‐Arsenault and Morrison‐Beedy 2001; DeBackere et al. 2008; Radford and Hughes 2015), that nurses who care for families after fetal loss may experience grief and emotional strain (Gandino et al. 2019; Hutti et al. 2016; Jonas‐Simpson et al. 2013; Shorey et al. 2017; Yenal et al. 2023), and that recurrent or involuntary pregnancy loss often creates substantial needs for treatment, support, and follow‐up (Dimitriadis et al. 2020; Fernández‐Basanta et al. 2020; Koert et al. 2019). Moreover, previous studies were not clearly describing the correlation between personal experiences of loss in pregnancy and maternity care professionals working as nurses and midwives in Jordan. It is crucial to investigate this issue since such health professionals might be supposed to care compassionately for pregnant women and cope simultaneously with grief, healing of their bodies, and concerns regarding future pregnancies. A scoping review of maternity healthcare professionals returning to work after a personal perinatal loss found no evidence from West Asian settings, underscoring the need for culturally situated research in Jordan (Musodza et al. 2023).

Jordan plays an important role in the context of this study, since it is an Arab and Muslim country, where marriage, starting families, and giving birth are considered desirable; moreover, religion has a particular view on fertility and conception, pregnancy, and infertility (Inhorn and Serour 2011; Serour 2008). Although these cultural and religious expectations may shape how pregnancy loss is discussed, they do not mean that women remain silent; rather, it is necessary to investigate their experiences in relation to the issues raised. Silence regarding pregnancy loss and the need for support has been noted in previous work on pregnancy loss and nursing care (Koert et al. 2019; Qian et al. 2022; Tektaş and Çam 2017), and Jordanian evidence also links social support with lower depression among pregnant women (Abuhammad et al. 2023). Accordingly, this study explored the lived experience of pregnancy loss among maternal nurses and midwives working in Jordan, with secondary attention to perceived effects on work and future pregnancy decisions.

## 2 Method

### 2.1 Design

The study used a descriptive phenomenological qualitative approach. This approach was selected because the purpose was to describe the essential meaning of participants' lived experience of pregnancy loss as expressed in their own accounts (Wojnar and Swanson 2007). The design focused on personal experience, embodied and emotional meaning, and the way pregnancy loss was interpreted within participants' clinical and cultural contexts.

### 2.2 Sample

The sample included 20 maternal nurses and midwives working in maternal health settings in Jordan who had experienced pregnancy loss. Inclusion criteria were: being a nurse or midwife working in a maternal ward or maternal health setting in Jordan; having experienced pregnancy loss; and being willing to discuss the experience in Arabic. No additional substantive exclusion criteria were applied beyond inability to meet these inclusion criteria; this decision was made to capture variation in loss histories while acknowledging in the limitations that time since loss and severity of loss varied across participants. The available demographic dataset did not separately record professional category; therefore, the precise nurse‐midwife split could not be reported and this issue is acknowledged in the limitations.

Participants were recruited through professional networks and participant referral using snowball sampling. Initial participants who met the eligibility criteria were invited to participate and then asked to refer other eligible individuals. Recruitment proceeded through approximately five referral waves. Snowball sampling was used to facilitate access to clinicians with a sensitive personal experience, not to achieve statistical representation. Efforts were made to include participants with varied backgrounds and experiences in order to capture a range of perspectives.

The recruitment sequence was invitation, information, electronic informed consent, verbal reconfirmation of consent, enrollment, and interview completion. Eligible participants received an electronic information and consent form that explained the study aim, potential risks and benefits, confidentiality, voluntary participation, and the right to withdraw at any time. All 20 eligible participants provided electronic informed consent before enrollment and verbally reconfirmed consent immediately before the interview. Twenty participants enrolled and completed interviews; no participant withdrew after enrollment. Interview scheduling and location were based on participant preference. Paper‐based written consent was not used because consent had already been documented electronically; the electronic consent and verbal reconfirmation procedures were included in the IRB‐approved study procedures.

### 2.3 Procedure

The interview guide included four open‐ended questions and additional prompts.

The interview guide was developed by the research team based on the study objectives and relevant literature on pregnancy loss and nursing care (Bansen and Stevens 1992; Fernández‐Basanta et al. 2020; Hutti et al. 2016; Nash et al. 2018; Radford and Hughes 2015). The guide was reviewed by the research team for clarity and relevance before data collection. Uncompleted template language was removed. The main interview domains included pregnancy history, physical and psychological effects, work effects, and decisions about future pregnancy.

Only individual semi‐structured interviews were conducted; no focus groups were held. Interviews were conducted by a female, PhD‐prepared member of the research team with prior training and experience in qualitative interviewing and professional familiarity with maternal nursing. She served as the interviewer and data collector and had no personal relationship with participants before data collection. No other person was present during the interviews besides the participant and interviewer. With participants' consent, all interviews were audio‐recorded to support accurate transcription and analysis. Recordings and transcripts were identified by study codes rather than participant names; access was limited to the research team, and the files were handled in accordance with the IRB‐approved confidentiality and data‐retention procedures. Where shared professional context could influence interpretation, this was addressed through reflexive notes and team discussion. No repeat interviews were conducted; each participant completed one interview.

The interviews typically lasted between 45 and 60 min. Data collection proceeded until the research team judged that sufficient thematic saturation had been achieved. Saturation was assessed by the interviewing researcher and discussed with the research team during preliminary analysis. Saturation was understood as the point at which no substantially new issues or perspectives were identified in successive interviews. Disagreements about saturation were handled through team discussion and return to the transcripts; new content was distinguished from confirmation when an interview introduced a meaning, experience, or contradiction that was not already represented in the developing themes. Saturation was considered to have occurred at approximately 13 interviews, after which additional data collection confirmed the recurrence of existing themes.

Participants worked in maternal health settings in Jordan, including health centers and government hospitals. Seventeen of 20 participants worked in health centers and 2 of 20 worked in government hospitals; 13 of 20 lived in cities and 7 of 20 lived in villages. Participant identifiers in the quotations consist of unique pseudonym numbers followed by age in years, for example P01(32). The author acknowledged her nursing academic background and prior engagement with the topic as potential influences on data collection and interpretation. Reflexivity was supported through written reflection before and after interviews, discussion of emerging interpretations with the research team, and repeated return to participants' words during analysis.

Participants were reminded before each interview that they could pause, decline to answer any question, or stop the interview without consequences. The authors explained why they were conducting the interviews and emphasized the importance of participants' views in enhancing understanding of pregnancy loss among maternal nurses and midwives.

### 2.4 Ethical Considerations

Ethical approval was obtained from the Institutional Review Board of The Hashemite University (HU‐IRB), approval reference No. 15/2/2023/2024, approved on 24 December 2023. The official IRB approval letter was provided with the submission. This study was conducted in accordance with the Declaration of Helsinki.

### 2.5 Reporting Method

This study was reported in accordance with the Consolidated Criteria for Reporting Qualitative Research (COREQ) 32‐item checklist for interviews and focus groups (Tong et al. 2007). A completed checklist is provided as File S1.

### 2.6 Data Analysis

Data from the interviews were analysed using Colaizzi's descriptive phenomenological method (Colaizzi 1978). First, transcripts were read repeatedly to obtain an overall sense of each account. Second, significant statements related to pregnancy loss were extracted. Third, meanings were formulated from these statements while staying close to participants' language. Fourth, formulated meanings were organized into theme clusters. Fifth, the theme clusters were integrated into an exhaustive description of the experience. Sixth, the exhaustive description was refined into the fundamental structure of the phenomenon. Seventh, all participants were invited to review their interview transcript and the research team's interpreted account for accuracy. Participants could identify inaccuracies, clarify meanings, or add information; any feedback received was checked against the transcript and considered before the thematic structure was finalized. A separate count of participants who returned additional corrections was not retained. Two researchers coded the data independently and then compared coding decisions; differences were resolved through discussion and reference to the transcripts. No dedicated qualitative data‐analysis software was used; coding and theme development were completed manually.

Data reliability was evaluated using Lincoln and Guba's (1985) trustworthiness criteria: credibility, dependability, confirmability, and transferability. Credibility was supported by inviting all participants to verify the accuracy of their transcripts and interpreted accounts, together with team comparison of interpretations across interviews. Dependability was enhanced by having another qualitative nursing researcher review the research process. Confirmability was supported through documentation of recruitment, data collection, coding, reflexive notes, and theme development. To support transferability, participant characteristics are reported in Table 1, including age, work setting, residence, pregnancy‐loss history, children, and years of nursing experience.

**TABLE 1:** Demographic characteristics of the participants (*N* = 20).

How old are you in years?	26–35	8	40.0
	36–45	6	30.0
	More than 45	6	30.0
How old is your husband in years?	18–25	2	10.0
	26–35	2	10.0
	36–45	7	35.0
	46–55	9	45.0
How old was the pregnancy when it was lost?	3 months	6	30.0
	4 months	3	15.0
	5 months	2	10.0
	6 months	2	10.0
	2 months	7	35.0
Do you have health insurance?	No	1	5.0
	Yes	19	95.0
What is your current employment status?	I do not work	1	5.0
	Employee at a health centre	17	85.0
	Employee at a government hospital	2	10.0
What is your monthly income in dinars?	401 to 600 JOD	11	55.0
	601 to 800 JOD	4	20.0
	Less than 400 Jordanian dinars	4	20.0
	More than 800 Jordanian dinars	1	5.0
What is your level of education?	Bachelor	5	25.0
	Diploma	15	75.0
What is your husband's educational level?	Primary or secondary	2	10.0
	Bachelor	9	45.0
	Diploma	7	35.0
	Graduate	2	10.0
What is your marital status?	Married	19	95.0
	Divorced	1	5.0
Do you have children?	No	2	10.0
	Yes	18	90.0
Where do you live?	Village	7	35.0
	City	13	65.0
Do you smoke?	No	17	85.0
	Yes	3	15.0
How many children do you have?	1	3	15.0
	2	2	10.0
	3	7	35.0
	4	4	20.0
	More than 4	2	10.0
	0	2	10.0
How many pregnancy losses have you had?	1	7	35.0
	2	6	30.0
	3	3	15.0
	4	1	5.0
	More than 4	3	15.0
Have you ever had the flu vaccine?	No	9	45.0
	Yes	11	55.0
Have you taken the Corona vaccine?	No	1	5.0
	Yes	19	95.0
How many years of nursing experience do you have?	6–10 years	3	15.0
	More than 10 years	17	85.0

## 3 Results

### 3.1 Demographic Data

A total of 20 maternal nurses and midwives participated in the study. The demographic dataset did not separately record professional category, so a precise nurse‐versus‐midwife count cannot be reported. The participants ranged in age from 26 to 55 years. Eight participants were 26–35 years old, six were 36–45 years old, and six were older than 45 years. Seventeen of 20 participants had more than 10 years of nursing experience. Seventeen worked in health centers, two worked in government hospitals, and one was not currently employed. Seven participants had experienced one pregnancy loss, six had experienced two losses, three had experienced three losses, one had experienced four losses, and three had experienced more than four losses. Eighteen participants had children, 13 lived in cities, and seven lived in villages. Refer to Table 1 below.

### 3.2 Themes

Four themes emerged from the data of this study: loss without warning; embodied loss: inseparable physical and psychological suffering; grieving while caregiving: negotiating work, disclosure, and peer support; and trying again with fear. Together, these themes describe the movement from unexpected loss, through bodily and emotional recovery, to the challenge of returning to maternal‐care work and considering future pregnancy.

#### 3.2.1 Loss Without Warning

Participants commonly described pregnancy as normal until a sudden sign, medical visit, or bodily change revealed that the pregnancy had ended. This theme reflects the disruption of trust in the body: participants moved from routine expectation to shock when loss occurred without warning. For example, P01(32y) said, “It was a normal pregnancy and things were normal and I had no complaints about anything. Suddenly the symptoms of fever disappeared. I went to the doctor in the fourth month and there was no pulse.” Moreover, P02(33y) said, “The first 2 months were excellent, and things were fine. By the third month, I had a Corona crisis and I was very tired of Corona. After I finished Corona, rested, recovered, and went back to work, during my check‐up in the fourth month there was no fetal pulse.” P03(30y) mentioned, “At first, it was good and things were going well. I was taking folic acid and eating well. Two months later, I suddenly started bleeding and my baby came out.” These accounts show that the loss was not experienced as a gradual clinical event but as an abrupt rupture between expected pregnancy progression and unexpected fetal absence.

#### 3.2.2 Embodied Loss: Inseparable Physical and Psychological Suffering

All 20 participants described pregnancy loss as affecting both physical and psychological health. Their accounts did not separate bodily recovery from grief; pain, bleeding, medical procedures, depression, and fear of recurrence were described as interwoven parts of the same experience. For example, P04(33y) said, “As for me, my psychology was greatly affected. I felt like I had lost my first child and had pain after a pregnancy loss, meaning pain like the pain of childbirth and labor, and I was afraid that the same thing would happen to me during the subsequent pregnancies.” Additionally, P05(39y) said, “I stayed several days after the operation, and then I had severe bleeding at home. I returned to the hospital and needed further treatment and blood transfusion. The experience was physically and psychologically exhausting.” Another participant, P06(42y), said, “I swear I fell into depression, which is not normal, but had it not been for a little religion, reading Qur'an, and sticking to the ritual, and without people's help, I would have really fallen into depression. I swear my condition was very difficult, and I did not think about getting pregnant until 2 years later.” The severe complication described by P05(39y) illustrates the range of physical severity in the sample and shows how medical trauma could intensify fear, mistrust, and delayed emotional recovery. P06(42y) also shows that religious practice was not peripheral; faith functioned as a source of psychological survival and meaning‐making after loss.

#### 3.2.3 Grieving While Caregiving: Negotiating Work, Disclosure, and Peer Support

Participants returned to workplaces that could remind them of pregnancy and birth, making grief difficult to separate from professional duties. Work effects included physical strain, leave‐taking, thoughts about changing workplace, continuing in the same role, support from colleagues, and reluctance to disclose personal grief.

##### 3.2.3.1 Changing, Continuing, or Adjusting Work

Some participants considered leaving or changing their workplace, whereas others continued working and used leave or workload adjustment instead. For example, P07(33y) said, “Oh, I thought about changing my place of work, because my work was tiring. I was working in the nurses' department, and it was a lot of physical effort to drag beds, strollers, and things like that, and chairs. So, frankly, I was thinking that I would leave my job or, for example, change my job because of this, what happened to me.” Additionally, P08(34y) reported, “I thought … I had to change my work system. It was tiring … But I took this step after I got pregnant and gave birth. After I gave birth, I resigned from my job.” A negative case was P05(39y), who did not plan to leave her job but relied on formal leave after the loss: “I did not plan to leave my job or change it. I took my leave with the committees … They gave me approximately more than a month and a half, and I was approved.” Together, these accounts indicate that pregnancy loss did not produce one uniform occupational response; instead, participants negotiated between bodily recovery, workplace demands, and the feasibility of changing jobs.

##### 3.2.3.2 Peer Support, Disclosure, and Silence at Work

The effectiveness of peer support was demonstrated by several respondents, but it was conditional on the participants' readiness to share their loss experience. For example, P04(33y) noted, “they supported me psychologically… they gave me a relief… after the cleaning and pregnancy loss, they started giving me work that I needed, which means that there will be less effort physical and psychological by all those supporting me.” In the same way, P08(34y) said, “of course they supported me psychologically and physically… in some situations when I needed work pressure they would do that work for me.” At the same time, another participant, P09(34y), showed a different pattern of peer relations: “I did not enjoy sharing my experience or listening to anyone's advice or talking to anyone … I do not enjoy talking about personal things.” The mentioned example proves that workplace support is not only connected with the presence of colleagues in the working place. It can depend on such factors as privacy, trust, and openness to talk about pregnancy loss.

#### 3.2.4 Trying Again With Fear: Future Pregnancy Between Persistence and Avoidance

Participants described future pregnancy decisions as a tension between desire for another pregnancy and fear that loss would happen again. Some continued trying to conceive, whereas others expressed avoidance or intense surveillance in later pregnancies. For example, P08(34y) said, “No, not at all. I mean, I said, if I had a pregnancy loss 30 times, I would continue to want … I would try.” P10(30y) reported, “The next time I got pregnant, I would follow up on the pregnancy from the beginning with a doctor. I was going to check on iron before pregnancy, take a stronger dose of folic acid and iron, and take stabilizers.” P02(33y) said, “I had no effect, I kept trying… After 4 months, my Lord honoured me and I got pregnant again.” Also, P11(43y) reported, “I started praying, oh Lord, for not having any new pregnancy. Very tiring experience.” Moreover, P12(44y) mentioned, “I became afraid of pregnancy, and every pregnancy I became afraid that the experience would be repeated … my follow‐up was not monthly. It was every 2 weeks.” These quotations indicate that future pregnancy was not simply accepted or rejected; it was re‐entered with heightened vigilance, prayer, fear, or avoidance depending on each participant's prior loss and recovery.

## 4 Discussion

This study contributes specific insights into experiences of pregnancy loss among maternal nurses and midwives in Jordan. While previous research has discussed changes in expectations related to pregnancy following perinatal loss (Côté‐Arsenault and Morrison‐Beedy 2001; DeBackere et al. 2008), experiences and needs of women after early pregnancy loss (Bansen and Stevens 1992; Radford and Hughes 2015), as well as grief reactions of nurses providing care to families whose newborn infant had died (Hutti et al. 2016; Jonas‐Simpson et al. 2013), this study makes a unique contribution by identifying how re‐entering a professional setting associated with pregnancy and childbirth could contribute to re‐experiencing grief in maternal nurses and midwives. This finding extends the scoping review by Musodza et al. (2023), which identified a need for further research on maternity healthcare professionals returning to work after personal perinatal loss and did not include evidence from West Asian settings.

The finding regarding bodily and emotional pain associated with pregnancy loss can be understood in relation to the existing literature suggesting that miscarriage is accompanied by both physical and psychological symptoms (Dimitriadis et al. 2020; El Hachem et al. 2017; Engelhard 2004; Kersting and Wagner 2012; Radford and Hughes 2015; Robinson 2014). In this case, participants spoke of physical manifestations of pain and bleeding, medical procedures, psychological issues such as depression and fear, as well as difficulty accepting the need to try for another pregnancy. The experience of miscarriage and the related feelings were influenced by the Islamic and cultural environment of Jordan with respect to motherhood and family formation and by religious coping strategies. This finding is consistent with literature emphasizing the need for treatment, support, and follow‐up after pregnancy loss (Koert et al. 2019; Tektaş and Çam 2017).

Workplace support emerged as a practical and emotional resource, especially when colleagues reduced physical workload or provided psychological support. This finding is consistent with studies showing the value of family, healthcare provider, and social support after pregnancy loss (Qian et al. 2022; Smith et al. 2020; Sun et al. 2018, 2020). However, the data also showed negative cases: some participants did not disclose the loss, and at least one participant did not plan to leave or change her job. This variability is consistent with evidence that staff responses to professionally encountered pregnancy and neonatal loss can include grief, emotional labor, distress, and different coping strategies (Gandino et al. 2019; McCreight 2005; Shorey et al. 2017; Wallbank and Robertson 2013). Therefore, recommendations should not assume that all clinicians want the same form of support. Support should be individualized and should include confidential leave, flexible workload adjustment, and access to trusted emotional support when desired by the clinician. These implementation considerations are consistent with Jordanian nursing evidence showing that organizational factors are major barriers to research utilization in clinical settings (Abuhammad et al. 2020), suggesting that bereavement‐support policies will require institutional support rather than individual motivation alone. Evidence from a Jordanian randomized clinical trial also indicates that guided imagery and progressive muscle relaxation can improve physical and emotional symptoms during stressful events (Alhawatmeh et al. 2022); such low‐burden approaches may be considered as supportive options, although their use after pregnancy loss among practicing nurses and midwives requires direct evaluation.

### 4.1 Implication of the Study

The findings support practice recommendations that are directly grounded in participants' accounts: confidential opportunities to talk, flexible workload adjustment after loss, nonjudgmental peer or supervisor support, and culturally sensitive acknowledgement of religious coping when the participant raises it. Broader bereavement‐care practices, such as private spaces or memory‐making, are discussed in the wider literature (Qian et al. 2023; Smith et al. 2020), but these should be distinguished from recommendations arising directly from the present data unless future analysis confirms that participants requested them.

### 4.2 Limitations

Limitations of this paper are discussed as follows. First of all, as a qualitative design, the sample size was small as it included only 20 participants that were selected using non‐probabilistic snowball sampling technique in Jordanian maternal healthcare services. Thirdly, there is a possibility of self‐selection bias since the sample involved the participants who were willing to talk about pregnancy loss and its effect on their lives and those who did not want to do that. Fourthly, the sample comprised Muslims that were chosen from Jordanian society; therefore, generalizability of the results to other countries or religions is questionable. Different periods since pregnancy loss, number of losses, and presence of any physical symptoms might affect memory. Workplace‐centered narratives included co‐workers, employers, and organizations; therefore, there is a possibility of social desirability bias in data collection. In addition, there is a possibility of distortion of data during translation of the quotations from Arabic into English language. All participants had an opportunity to participate in data checking and interpretation; however, there was no record on the number of people who sent back their corrections. Finally, the demographic data set did not distinguish between nurses and midwives; thus, it is impossible to analyse these occupations separately. The study did not consider different methods of intervention (e.g., guided imagery, progressive muscle relaxation); thus, further empirical testing of these techniques is required.

## 5 Conclusion

This study highlighted the experience of pregnancy loss among maternal nurses and midwives through their embodiment, grief, culture and religion, and return to workspaces that involve pregnancy and childbirth. Participants reported sudden pregnancy loss, a combination of body and mind pain, different reactions from their working environment, and decisions related to future pregnancies. Further research needs to compare nurses and midwives in different environments of Jordan as well as study the influence of role, time of loss, complications, and religious coping on pregnancy losses.

## Author Contributions

**Sawsan Abuhammad:** validation, investigation, writing – original draft, funding acquisition, conceptualization, formal analysis, project administration, visualization, writing – review and editing, data curation, supervision. **Shaher Hamaideh:** methodology, validation, visualization, formal analysis, project administration, funding acquisition, supervision, resources, investigation. **Hossam Alhawatmeh:** investigation, funding acquisition, writing – original draft, validation, visualization, writing – review and editing, project administration, formal analysis, data curation, supervision, resources. **Rowaida AlMaaitah:** supervision, formal analysis, project administration, validation, visualization, investigation, funding acquisition. **Heba H. Hijazi:** funding acquisition, writing – original draft, visualization, writing – review and editing, formal analysis, project administration, data curation, supervision, resources. **Maryam K. El‐zubi:** validation, writing – review and editing, formal analysis, writing – original draft, methodology, visualization, supervision. **Amat Al‐Khaleq Mehrass:** validation, investigation, formal analysis, resources, project administration, writing – review and editing. **Ahlam Hamaidi:** resources, formal analysis, project administration, visualization, writing – review and editing, funding acquisition, writing – original draft. **Vidya Seshan:** investigation, funding acquisition, writing – original draft, formal analysis, project administration, supervision, resources, visualization. **Nabeel Al Yateem:** validation, writing – review and editing, investigation, funding acquisition, writing – original draft, formal analysis, project administration, supervision, visualization, data curation.

## Funding

The authors have nothing to report.

## Ethics Statement

This study was approved by Hashemite University.

## Conflicts of Interest

The authors declare no conflicts of interest.

## Supporting information


**File S1:** Completed COREQ Checklist.

## Data Availability

The data that support the findings of this study are available on request from the corresponding author. The data are not publicly available due to privacy or ethical restrictions.
