# Supplementary material for: Experience of Pregnancy Loss Among Maternal Nurses and Midwives: A Phenomenological Study
Source: Nurs Open. 2026 Oct 12;13(10):e70892. doi: 10.1002/nop2.70892 (PMC13658935; doi:10.1002/nop2.70892)
Supplement: Supplementary file 1 [file NOP2-13-e70892-s001.docx]

Supplementary File 1: Completed COREQ Checklist

**Experience of Pregnancy Loss Among Maternal Nurses and Midwives: A Phenomenological Study**

**Reporting guideline selected:** Consolidated Criteria for Reporting Qualitative Research (COREQ), because the study used individual semi-structured interviews within a descriptive phenomenological qualitative design.

**Completion convention:** Every item is completed. “Not reported” identifies information that is not stated in the supplied manuscript; no unsupported methodological details were invented. Page numbers refer to the revised highlighted manuscript.

# COREQ 32-item checklist

| **No.** | **Item** | **Guide question/description** | **Where reported in revised manuscript / completion status** |
| --- | --- | --- | --- |
| **Domain 1: Research team and reflexivity** | | | |
| 1 | Interviewer/facilitator | Which author/s conducted the interview or focus group? | p. 6: Interviews were conducted by members of the research team. Individual author names are not specified. |
| 2 | Credentials | What were the researcher’s credentials? | Not reported in the supplied manuscript. |
| 3 | Occupation | What was their occupation at the time of the study? | p. 6: Nursing academic background and professional familiarity with maternal nursing are reported; a precise occupational title is not stated. |
| 4 | Gender | What was the researcher’s gender? | Not reported for the interviewer(s). |
| 5 | Experience and training | What experience or training did the researcher have? | p. 6: Training and prior experience in qualitative interviewing are reported. |
| 6 | Relationship established | Was a relationship established before study commencement? | p. 6: The interviewer had no personal relationship with participants before data collection. |
| 7 | Participant knowledge of interviewer | What did participants know about the researcher? | p. 7: Authors explained why the interviews were being conducted and the importance of participants’ views. |
| 8 | Interviewer characteristics | What characteristics/biases/assumptions were reported? | p. 6: Nursing academic background, professional familiarity, reflexive notes, and team discussion are reported. |
| **Domain 2: Study design** | | | |
| 9 | Methodological orientation and theory | What methodological orientation underpinned the study? | pp. 4 and 7: Descriptive phenomenology and Colaizzi’s seven-step method. |
| 10 | Sampling | How were participants selected? | pp. 4-5: Snowball sampling through professional networks and participant referral. |
| 11 | Method of approach | How were participants approached? | pp. 4-5: Initial invitations through professional networks followed by participant referral. |
| 12 | Sample size | How many participants were in the study? | pp. 4 and 8: 20 maternal nurses and midwives. |
| 13 | Non-participation | How many refused or withdrew, and why? | p. 5: Twenty eligible participants were invited, consented, enrolled, and completed interviews; none withdrew after enrollment. |
| 14 | Setting of data collection | Where were data collected? | pp. 1 and 5: Individual interviews were conducted in private locations selected according to participant preference; specific site types are not reported. |
| 15 | Presence of non-participants | Was anyone else present? | Not reported in the supplied manuscript. |
| 16 | Description of sample | What important sample characteristics were reported? | pp. 6 and 8; Table 1, pp. 19-20: age, work setting, residence, pregnancy-loss history, children, nursing experience, and other demographics. |
| 17 | Interview guide | Were questions/prompts provided and pilot tested? | p. 5: Four open-ended questions and prompts are described; domains and team review for clarity/relevance are reported. The complete guide and formal pilot testing are not reported. |
| 18 | Repeat interviews | Were repeat interviews conducted? | Not reported in the supplied manuscript. |
| 19 | Audio/visual recording | Was recording used? | Not reported in the supplied manuscript. |
| 20 | Field notes | Were field notes made? | p. 6: Reflexive notes before and after interviews are reported; data-collection field notes are not explicitly reported. |
| 21 | Duration | What was the duration of interviews? | p. 6: 45-60 minutes. |
| 22 | Data saturation | Was saturation discussed? | p. 6: Saturation definition, assessment, team discussion, and approximate occurrence at 13 interviews are reported. |
| 23 | Transcripts returned | Were transcripts returned for comment/correction? | pp. 7-8: Participants were invited to review transcripts and the accuracy of the interpreted account. |
| **Domain 3: Analysis and findings** | | | |
| 24 | Number of data coders | How many coders coded the data? | p. 7: Two researchers coded independently and resolved differences through discussion and transcript review. |
| 25 | Description of coding tree | Was a coding tree described? | pp. 7-8: Theme clustering and the four final themes are described; a separate coding tree is not provided. |
| 26 | Derivation of themes | Were themes identified in advance or derived from data? | pp. 7-8: Significant statements, formulated meanings, theme clusters, and final themes were derived through Colaizzi’s method. |
| 27 | Software | What software was used to manage data? | Not reported in the supplied manuscript. |
| 28 | Participant checking | Did participants provide feedback on findings? | pp. 7-8: Transcript and interpreted-account accuracy checking are reported; the limitations note that thematic member checking was not fully reported. |
| 29 | Quotations presented | Were participant quotations used and identified? | pp. 9-12: Quotations are presented with participant pseudonym numbers and ages. |
| 30 | Data and findings consistent | Was there consistency between data and findings? | pp. 9-12: Quotations are linked directly to each theme and interpretive statement. |
| 31 | Clarity of major themes | Were major themes clearly presented? | pp. 8-12: Four major themes are named, organized, and described. |
| 32 | Clarity of minor themes/diverse cases | Were diverse cases or minor themes discussed? | pp. 11 and 13: Negative cases and variation in disclosure, peer support, and occupational responses are explicitly discussed. |

**COREQ source:** Tong A, Sainsbury P, Craig J. Consolidated criteria for reporting qualitative research (COREQ): a 32-item checklist for interviews and focus groups. International Journal for Quality in Health Care. 2007;19(6):349-357. doi:10.1093/intqhc/mzm042.
